# Orthodontic Tooth Movement with Clear Aligners

**DOI:** 10.5402/2012/657973

**Published:** 2012-08-14

**Authors:** Carl T. Drake, Susan P. McGorray, Calogero Dolce, Madhu Nair, Timothy T. Wheeler

**Affiliations:** ^1^Private Practice 310 Susan Drive, Suite 1, Normal, IL 61761, USA; ^2^Department of Biostatistics, University of Florida, Box 117450, FL 32611, USA; ^3^Department of Orthodontics, School of Advanced Dental Sciences, College of Dentistry, University of Florida, Gainesville, FL 32610, USA; ^4^Department of Oral and Maxillofacial Diagnostic Sciences, College of Dentistry, University of Florida, Gainesville, FL 32610, USA

## Abstract

Clear aligners provide a convenient model to measure orthodontic tooth movement (OTM). We examined the role of in vivo aligner material fatigue and subject-specific factors in tooth movement. Fifteen subjects seeking orthodontic treatment at the University of Florida were enrolled. Results were compared with data previously collected from 37 subjects enrolled in a similar protocol. Subjects were followed prospectively for eight weeks. An upper central incisor was programmed to move 0.5 mm. every two weeks using clear aligners. A duplicate aligner was provided for the second week of each cycle. Weekly polyvinyl siloxane (PVS) impressions were taken, and digital models were fabricated to measure OTM. Initial and final cone beam computed tomography (CBCT) images were obtained to characterize OTM. Results were compared to data from a similar protocol, where subjects received a new aligner biweekly. No significant difference was found in the amount of OTM between the two groups, with mean total OTM of 1.11 mm. (standard deviation (SD) 0.30) and 1.07 mm. (SD 0.33) for the weekly aligner and biweekly control groups, respectively (*P* = 0.72). Over eight weeks, in two-week intervals, material fatigue does not play a significant role in the rate or amount of tooth movement.

## 1. Introduction

Research of orthodontic tooth movement (OMT) using clear aligners is limited. Most of the literature consists of case reports, editorials, or articles written by authors with biases. There have been few evidence-based attempts to describe the type of OTM resulting from treatment with clear aligners. Conventional thinking suggests that the movement is mostly uncontrolled tipping, with the center of rotation located between the center of resistance and the apex of the tooth. The center of resistance of a single-rooted tooth has been reported to be on the long axis of the tooth between one-third and one-half of the root length apical to the alveolar crest [1].

Clinical trials of aligners have examined the entire course of treatment. Bollen et al. [2] report on the comparisons of two types of material (hard, soft) and two activation frequencies (1 week, 2 week). Fifty-one subjects were randomized to the four groups and evaluated for the primary endpoint: completion of initially prescribed aligner series. More subjects completed the initial series (37% versus 21%) in the two-week activation group, and no difference due to the fact that material was detected. Clements et al. [3] examined the end-of-study models of the above subjects, focusing on weighted Peer Assessment Ratings (PAR scores), PAR components, average incisor irregularity, and papillary bleeding scores. No significant differences were observed between the four groups. Kravitz et al. [4] reviewed results of 37 patients (401 teeth) treated with clear aligners and compared predicted tooth movement to achieved tooth movement. The mean accuracy over all types of movement was only 41%. Many subjects who begin clear aligner treatment deviate from the programmed progression of aligners and require reevaluation, midcourse correction, and/or use of fixed appliances to achieve treatment goals. A better understanding of the mechanics of tooth movement using aligners could lead to more appropriate selection of patients, better sequencing of tooth movement stages, and more efficient treatment. 

Duong and Kuo [5] compared the load deflection rates (LDR) of 0.017 × 0.017′′ stainless steel (SS) and Nickel Titanium (NiTi) wires versus 0.030 mm. polyurethane material over a 0–10% range of strain in vitro. The LDR of the polyurethane was greater than the NiTi wire but less than the stainless steel wire. Therefore, with a given amount of deflection, the aligner should deliver a lower initial level of force than the SS wire. Additional studies relating force levels to aligners would be beneficial, including clinical studies addressing deformation of aligners in the oral environment over time.

A randomized and controlled clinical trial was performed at the University of Florida in 2005, evaluating the safety, tolerability, and efficacy of recombinant human relaxin during OTM using clear aligners [6]. This study found no significant difference between the treatment and control groups concluding that the use of relaxin did not affect the rate or amount of OTM. However, several interesting observations were noted including the following: (1) more OTM occurred during the first week than during the second week of aligner wear for each two-week prescription cycle, (2) the full prescription of the aligners was not expressed, and (3) OTM was highly variable among individuals. These observations have clinical implications and require further research.

Currently patients wear each aligner for two weeks, although there is little evidence to support this. Further, the role of in vivo aligner degradation on OTM is unknown, but it likely results in a decrease in the magnitude of forces transferred to teeth over time. If material fatigue inhibits aligners from fully expressing their potential or prescription, we hypothesize that replacing each aligner after one week with a new aligner with an identical prescription may increase tooth movement. Patients and clinicians could benefit from a better understanding of the impact of appliance degradation and fatigue on OTM. The purpose of this study is to test whether total OTM over the course of 8 weeks differs, comparing the standard protocol of a new aligner and prescription every two weeks (historical data) to standard protocol plus a new aligner (same prescription) received for even weeks. In addition, preliminary analyses will examine correlation between impression-based tooth movement and CBCT variables representing tooth morphology and bone quality.

## 2. Methods

### 2.1. Study Design

This study was a prospective single-center clinical trial involving subjects with minor incisor malalignment, who were otherwise healthy and intended to undergo comprehensive orthodontic treatment. The design for this study was similar to the previous relaxin aligner study [6]. The 37 subjects who participated in the relaxin study served as the retrospective biweekly control group. Both studies were approved by the University of Florida Institutional Review Board for the Protection of Human Subjects.

Sixteen subjects were enrolled and one dropped out (moved out of area) during the screening process prior to initiating treatment, resulting in a sample of 15 adult subjects (6 males and 9 females) in the weekly aligner group. Subjects were in good health, were not chronic users of NSAIDS or steroid medications, did not smoke within the last 6 months, were not pregnant, and had appropriate dental characteristics (adult dentition, at least one upper maxillary central incisor with sufficient space to allow AP movement of 2 mm., normal pulp vitality, gingival attachment, papillary bleeding and pocket depth, and no active caries). 

The right or left maxillary central incisor was selected as the target tooth based on this tooth not being blocked out by the adjacent teeth. In the event that either tooth would qualify, one was chosen at random.

During the study, subjects were provided with four maxillary aligners, each programmed to move the target tooth 0.5 mm. every two weeks for a total programmed tooth movement of 2.0 mm. Only bodily movement of the single target tooth in the A-P dimension was programmed (no intrusion, extrusion, or rotation). In addition, four duplicate aligners were fabricated for the replacement of the delivered aligner at the beginning of each odd-numbered week. Therefore, new aligners were dispensed weekly. This is in contrast to the retrospective biweekly control group, where subjects wore only four aligners, each for a period of two weeks for a total programmed tooth movement of 2.0 mm. The final time point for data collection, marking the end of the study, was at week 8 

All study subjects were instructed to wear the aligners full time. However, they were allowed to remove the appliance when eating, drinking, or brushing their teeth.

### 2.2. Enrollment and Study Participation

To determine subject eligibility, two screening visits were required. The purpose of the initial screening visit was to identify potential subjects with malocclusions needing minor incisor alignment and to eliminate those with medical conditions or intraoral problems that were exclusionary. At the second screening, subject's eligibility was confirmed and initial records were collected. The following procedures were performed: (1) PVS impressions, (2) intraoral and extraoral photographs, and (3) CBCT imaging. 

At the first study visit, week 0, the first aligner was delivered with instructions to wear full time except while eating, drinking, and brushing. The acceptable visit window for weeks 1 through 8 was ± one day, and all 15 treatment subjects successfully satisfied this requirement. At subsequent weekly visits maxillary PVS impressions as well as frontal and occlusal photographs were taken and the next aligner was delivered. At the final study visit, week 8, maxillary PVS impressions and final photographs were taken as well as CBCT imaging of the maxilla. Note that the biweekly control group did not undergo CBCT imaging.

### 2.3. Collection of Data

Weekly digital models were fabricated from PVS impressions. Models from weeks 1 through 8 were then superimposed with the initial model from screening 2, according to the best fit of the posterior teeth, using Align Technology's Tooth Measure software, version 2.3. The centroid of the clinical crown of the target tooth was established, and the amount of A-P and vertical OTM of the target tooth was then measured for each time point relative to baseline. The A-P axis was determined by the direction of programmed OTM in ClinCheck. Examiner 1 (an orthodontic resident) measured the models of the 15 treatment subjects, and examiner 2 (a 3rd year dental student) measured the 37 biweekly control subjects. 

**Figure 1:**
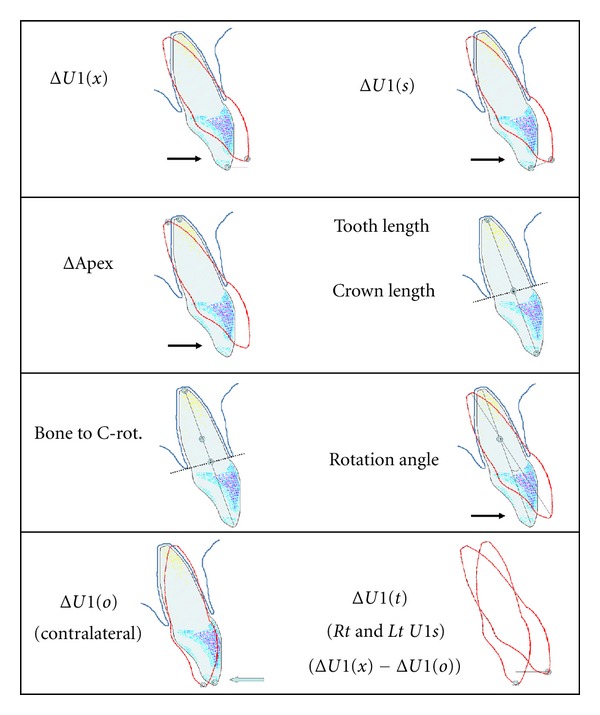
Superimposed CBCT measurements. Blue is initial and red is final. Δ*U*1(*x*) refers to the distance between lines drawn through the midpoint of the incisal edges of the superimposed target tooth perpendicular to the A-P axis (the plane of prescribed tooth movement). Δ*U*1(*s*) is the length of the line connecting the midpoint of the incisal edges of the superimposed target tooth. ΔApex refers to the length of a line connecting the change in apex of the superimposed target tooth. *Rotation angle* is the angle created by the intersection of lines drawn from the midpoint of the incisal edge to the apex of the target tooth. The apex of this angle is considered the *center of rotation*. *Tooth length* refers to the distance from the midpoint of the incisal edge to the apex of the target tooth from the initial X-ray. *Crown length* is the portion of the tooth length that is coronal to the bone. *Bone to C-rot*. is the section of tooth length between the center of rotation and a line connecting the most coronal aspect of the faciolingual crestal bone. Δ*U*1(*o*) refers to the A-P change in the midpoint of the superimposed incisal edge of the opposite central incisor, the one that was not the target tooth. Δ*U*1(*t*) refers to the distance between midpoint of the superimposed incisal edge of the contralateral central incisor, to the midpoint of the incisal edge of the target tooth.

CBCT images were obtained at screening and in week 8. Using Anatomage's InVivoDental software, version 4.1, the orientation of these images was adjusted by examiner 1 to standardize the A-P axis with the corresponding digital models. Initial and final images were superimposed, registered on the curvature of the palate and best fit of maxillary bony structures. Multiple measurements were obtained and are illustrated in Figure 1.

A fractal analysis score [7] was calculated from the CBCT for each subject in the weekly aligner group, which was used to determine the quality of the bone. CBCT slices through comparable planes were obtained across all subjects. Images were subjected to histogram equalization using a reference image, and a region of interest (ROI) adjacent to the apex of the target tooth was selected for use on all images. Fractal analyses were done for each of the different ROIs using the power spectrum method employed by the TACT workbench. Thirty-two bit complex floating point representations of the ROIs were cropped, and subject to 2D Fast Fourier transform (FFT), followed by plotting the log of the magnitude versus frequency component that was generated by the FFT. A regression line was fit to this plot, and the slope of this line was used to generate a fractal dimension (FD) for each of the ROIs. The higher the FD, the higher the morphological complexity at the ultrastructural level of bone. Analyses of FD have been correlated with the strength of bone in previously reported studies [8]. 

### 2.4. Calibration

Examiners 1 and 2 were trained to use the Tooth Measure software on the same day, and the following measurement protocol was agreed upon: (1) allow the software to ignore teeth according to its “statistical filtering” protocol, (2) always ignore teeth immediately adjacent to the target tooth as well as the target tooth itself, and (3) instruct Tooth Measure to superimpose the models according to the best fit of the remaining teeth. Interexaminer reliability was determined after separately measuring six randomly selected subjects from the 2005 study, with eight superimpositions per subject. Results were identical between Examiners 1 and 2. 

For the CBCT data, Examiner 1 remeasured the following variables on a different day to determine reproducibility: distance between midpoints of incisal edges of the superimposed target tooth, rotation angle, tooth length, and crown length. The intraclass classification coefficient estimates (ICC) of Fliess [9, 10] was determined, using R software (R Development Core Team, Vienna, Austria) to quantify the strength of relationship between the duplicate measurements. ICC values ranged from 0.90 to 0.99, which demonstrated excellent reliability.

### 2.5. Statistical Considerations

The sample size was based on the ability to detect difference in 8-week tooth movement between the weekly aligner group and the historical biweekly control group. Based on the variance estimate from the biweekly control group and using a two-sided *t*-test with level of significance 0.05, we had adequate (0.80) power to detect a difference in mean tooth movement of 0.22 mm. Using only the weekly aligner group, we have adequate power to detect correlations of 0.43 or larger, between tooth movement and the CBCT variables. (CBCT imaging was not done on the historical controls.)

The amount of A-P OTM of the target tooth from baseline to week 8 was assessed for the 15 weekly aligner subjects that completed the study. Model data from the sample was compared with data from the biweekly control group with a sample size of 37. The null hypothesis of no difference in OTM from baseline to week 8 between the biweekly control group and weekly aligner group was tested using a two-sample *t*-test with a level of significance set at 0.05. Mixed model analysis was used to test the difference between the first week of any given two-week interval versus the second week and differences within treatment group over the four two-week cycles. Summary statistics were calculated for the measures of tooth movement and morphology based on model and CBCT assessments. Spearman correlation coefficient estimates were used to assess relationships between these variables. SAS (Version 9.1.3, SAS Institute Inc., Cary NC) statistical software was used to conduct the analysis. 

## 3. Results

Demographic information for the weekly aligner group is compared with the biweekly control group in Table 1. Subjects ranged from age 18 to 40 (mean age 25.1), 67% were female, and the groups did not differ significantly with respect to age, sex, or race.

**Figure 2:**
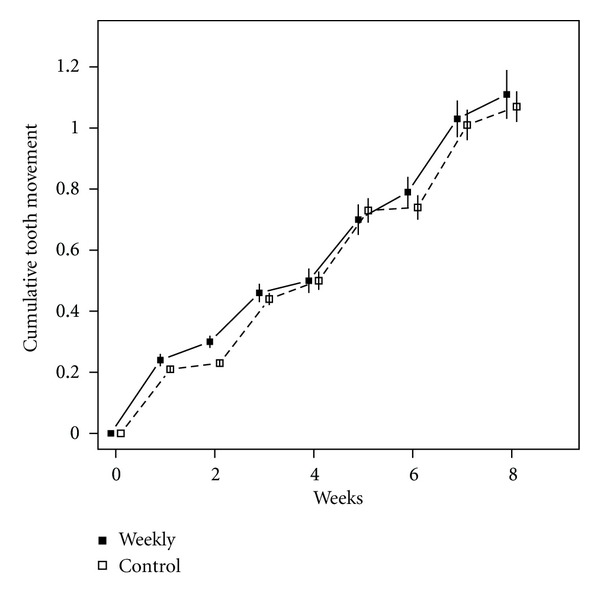
Cumulative tooth movement for each group by week. Mean and standard error bars are shown and lines have been offset for clarity.

Results from model measurements and comparisons between groups and time periods are summarized in Table 2, and a comparison of weekly mean values for the treatment and control groups is illustrated in Figure 2. No overall difference in OTM was detected between the groups, with mean total OTM of 1.11 mm. (standard deviation (SD) 0.30) and 1.07 mm. (SD 0.33) for the weekly aligner and biweekly control groups, respectively (*P* = 0.72). Also, no difference was detected in weekly OTM of the weekly aligner versus biweekly control groups overall (*P* = 0.812) or between any two-week prescription cycle for the weekly aligner and biweekly control groups (*P*'s = 0.176 and 0.297). However, 4.4 times more OTM occurred during the first week than the second week of aligner wear (*P* < 0.001) for the combined groups, considering all two-week periods.

Summary statistics for age and CBCT measures and correlations with the model-based measure of tooth movement are displayed in Table 3. Measurements from superimposed CBCT images confirmed that the target tooth experienced uncontrolled tipping. The center of rotation, on average, was located a distance of 41% of the root length apical to the faciolingual crestal bone. The incisal edge of the target tooth moved more than the centroid of the clinical crown in all cases, with a mean of 1.56 mm. for Δ*U*1(*x*) compared with 1.10 mm. measured from the centroid. Δ*U*1(*s*) had a mean of 1.63 mm, compared with the mean Euclidian mean value of 1.11 mm. measured from the centroid of the clinical crown on the models. The apex of the target tooth moved in the opposite direction with a mean of −0.73 mm. The contralateral central incisor experienced a loss of anchorage measured from the incisal edge, with a mean OTM of −0.28 mm. Mean fractal dimension determined from the CBCT's of the 15 weekly aligner subjects was 1.71 ± SD 0.20.

Due to small sample size, we had limited power to detect significant correlations. As expected, the model-based measure of tooth movement was highly correlated with the CBCT measures of movement. Correlation estimates of magnitude 0.40 or greater were identified for age, tooth length, and root length.

## 4. Discussion

This study replicated previous findings that the vast majority of OTM during any 2-week aligner prescription cycle occurs during the first week of the cycle. The target tooth in this eight-week study did not undergo the classic cycle of tooth movement described by Krishnan and Davidovitch [11]. This may be due to the two-week activation cycle or the inability of the removable polyurethane aligners to produce a continuous force. Due to incomplete expression during the previous seven weeks, the amount of target tooth activation after delivery of the eighth aligner was in excess of 1 mm, according to model data. This likely resulted in the tooth feeling a greater force during the last week than after delivery of the first and second aligners, when the amount of activation was 0.5 mm. or less. However, significantly less OTM occurred at week 8 than week 1, and there was no significant difference in the amount of OTM observed at week 8 and week 2. This suggests that the discrepancy in the amount of OTM achieved during the first and second week of each prescription cycle cannot be explained by force magnitude. Our finding that the use of new duplicate aligners did not increase the amount of OTM further supports this. 

Although bodily protraction of the target tooth was programmed, uncontrolled tipping resulted, which has clinical implications. More specifically, the result will be different from the programmed amount, and aligner tracking and retention may be negatively affected. When clinicians attempt to move maxillary incisors in the A-P dimension with aligners, allowing for vertical changes of the incisal edges may make treatment more predictable, which could reduce the need for midcourse modifications and make treatment more efficient. The amount of relative intrusion and extrusion to program can be determined by estimating the location of the center of rotation of the teeth (determined from our data to be approximately 41% of the root length apical to the faciolingual crestal bone). 

According to model data, the full prescription was not expressed in any of the 52 subjects. In fact, the mean OTM for both groups was only 1.1 mm, or 55% of the prescription. It is important to remember that the prescribed amount of OTM was at least twice the maximum rate per aligner currently prescribed for patient treatment. It is possible that a greater percentage of the prescription will be achieved if the maximum two-week activation was decreased to 0.25 mm. or less instead of 0.5 mm. 

 The discrepancy between the amount of OTM prescribed and that achieved may be partially explained by the method of measurement used in this study as well as the uncontrolled tipping that occurred. The largest amount of OTM recorded from baseline to week 8 from model data was 1.44 mm, or 72% of the prescription. This same subject had 1.98 mm. of OTM from baseline to week 8 when measured from the incisal edge of the target tooth of superimposed CBCT images. Given this information, one would not expect 100% of the prescription at the centroid of the crown to be fulfilled since OTM at the incisal edge was already fully achieved. 

One must also consider anchorage loss of the contralateral central incisor when interpreting this data. The prescribed protraction of the target tooth relative to the contralateral central incisor at week 8 was two mm. for each subject, and the difference between Δ*U*1(*o*) and Δ*U*1(*x*), or Δ*U*1(*t*) indicates that a mean of 1.85 mm. of this two mm. distance was actually fulfilled, an average of 92.3% of the prescription. In addition, eight of the 15 subjects showed a total OTM greater than 1.9 mm, which indicates that OTM at the incisal edge of these subjects was nearly fully expressed. 

Correlations between model-based OTM measures and CBCT measures were high, for assessing tooth movement over the eight-week time period. This suggests that model-based estimates may be used to evaluate incremental tooth movement. A better understanding of the biological process may be obtained if measurements that are more frequent were obtained over the course of the two-week period. Analysis based on von Mises strains during aligner treatment suggest that most of the movement would occur within the first 24 hours of placement [12]. Additional measurements over a two-week period, midtreatment, would further quantify the pattern of OTM with clear aligners. 

Our eight-week movement study of a single tooth is somewhat artificial, as teeth are programmed to move over intermittent periods of time and not in isolation. However, our results are comparable to the total treatment efficiency results reported by Kravitz et al. [4]. They reported a mean overall accuracy (compared to programmed movement) of 41%, with 48.5% obtained for the maxillary central incisor.

The measurement method explains a portion of the discrepancy in model data between programmed and actual OTM. However, tremendous variation was reported among subjects, and several target teeth did not achieve their full prescription at the incisal edge. Some variability between the subjects may be explained by lack of compliance. However, from a biologic perspective, it is likely that a subset of subjects did not have the capacity to keep up with the prescribed rate of OTM. According to Krishnan and Davidovitch [13], several systemic factors can influence rates of OTM, and some were specifically excluded from this study. Others that were not controlled, such as sex, age, bone quality, tooth length, and the location of the center of resistance (determined by root length, root width, and bone height) [1, 14] are likely involved.

The result of the exploratory data analysis assessing correlation of several biologic variables with OTM was inconclusive due to limited sample size. Some trends were noted, however, and future research with larger numbers of participants will be necessary to explore these findings. 

There was considerable variability of OTM in this study, which is a problem that practicing orthodontists often encounter. Improving the ability to identify patients who are unlikely to respond well to treatment would be beneficial for the profession and should be a focus of ongoing research. Regarding OTM using clear aligners, methods of altering treatment to compensate for patients who may not respond as well to OTM include: (1) establishing realistic expectations, (2) spreading treatment over additional aligners, thus decreasing the programmed rate of tooth movement, and (3) programming overcorrection. 

## 5. Conclusions

This single-center clinical trial examined OTM using clear aligners. No significant difference over an 8-week time period was found in the amount of OTM between those who wore the same aligner for two weeks compared to those who changed to a new duplicate aligner after one week. Therefore, the reduction in the amount of OTM seen during the second week of aligner wear was likely not due to material fatigue. The method of tooth movement measurement can affect the interpretation of results, especially when uncontrolled tipping occurs. Other variables that could affect tooth movement such as age, sex, root characteristics, and bone quality were examined and suggest areas for future investigation.

## 6. Clinical Relevance

Treatment using clear aligners is becoming increasingly common in orthodontics. A better understanding of how tooth movement is achieved may lead to treatments that are more efficient. We examined the potential role of material fatigue over four two-week time periods and did not detect any difference in tooth movement between a control group and a group that received a new aligner of the same prescription after one week of the two-week prescribed wear time for each aligner. The role of uncontrolled tipping and loss of anchorage complicate the progression of programmed aligners. Further evaluation of patient characteristics, such as age, bone quality, and tooth morphometrics could aid in aligner treatment planning. 

## Figures and Tables

**Table 1:** Comparison of demographics of weekly aligner versus biweekly control groups.

Age	*N *	Mean	SD	Min	Max	*P* value
Weekly aligner	15	25.5	4.8	20.5	34.9	0.50*
Biweekly control	37	26.66	5.12	18.56	40.48	

Sex	Female	Male	% Female			

Weekly aligner	9	6	60%			0.52**
Biweekly control	26	11	70%			

Race	White	Black	Asian	Hispanic	Pac. Island.	

Weekly aligner	8 (53%)	2 (12%)	3 (20%)	1 (7%)	1 (7%)	0.08***
Biweekly control	28 (76%)	5 (14%)	1 (3%)	3 (8%)	0	

*Wilcoxon rank sum test.

**Fisher exact test.

***White versus nonwhite Fisher exact test.

**Table:** (a) Mixed modeling comparing mean OTM per week from baseline to week 8 for weekly aligner versus biweekly control groups

Group	Mean/Wk ± SD	*P* value
Weekly aligner Biweekly control	0.14 ± 0.11	0.812
	0.14 ± 0.15	

**Table:** (b) Mixed modeling comparing the mean magnitude of OTM per week expressed during each two-week prescription cycle

Group	Interval	Mean/Wk ± SD	*P* value
Weekly aligner	Week 1-2	0.15 ± 0.11	0.176
	Week 3-4	0.10 ± 0.09	
	Week 5-6	0.15 ± 0.11	
	Week 7-8	0.16 ± 0.13	

Biweekly control	Week 1-2	0.12 ± 0.11	0.297
	Week 3-4	0.13 ± 0.13	
	Week 5-6	0.13 ± 0.17	
	Week 7-8	0.16 ± 0.19	

**Table:** (c) Mixed modeling comparing OTM during the first week versus second week for the weekly aligner and biweekly control groups, both separately and combined

Group	Interval	Mean/Wk ± SD	*P* value
Weekly aligner	1st week	0.21 ± 0.09	<0.0001
	2nd week	0.07 ± 0.08	
Biweekly control	1st week	0.23 ± 0.13	<0.0001
	2nd week	0.04 ± 0.11	

Total	1st week	0.22 ± 0.12	<0.0001
	2nd week	0.05 ± 0.10	

**Table 3:** Descriptive statistics and correlations between CBCT measurements and model-based tooth movement (*n* = 15, weekly aligner group).

Variable	Mean	SD	Min	Max	Spearman correlation	*P* value
					with model tooth movement	
Age	25.50	4.80	20.50	35.90	−0.46	0.08
Δ*U*1(*x*)	1.56	0.38	0.80	2.02	0.90	<0.0001
Δ*U*1(*s*)	1.63	0.40	0.80	2.09	0.86	<0.0001
ΔApex	−0.73	0.26	−1.32	−0.39	−0.72	0.0023
Tooth length	24.87	2.02	21.67	30.32	−0.42	0.12
Crown length	12.27	0.74	10.84	13.27	−0.17	0.55
Root length	12.60	1.74	10.56	17.74	−0.40	0.14
Crown/root ratio	0.99	0.12	0.71	1.23	0.12	0.67
Bone to C-rot	5.14	1.25	2.89	7.70	−0.10	0.72
Δ*U*1(*o*)	−0.28	0.16	−0.52	0.00	0.25	0.38
Δ*U*1(*t*)	1.85	0.36	1.08	2.40	0.70	0.0036
Fractal dimension	1.71	0.20	1.37	2.00	0.25	0.36

## References

[1] Burstone CJ, Pryputniewicz RJ (1980). Holographic determination of centers of rotation produced by orthodontic forces. *American Journal of Orthodontics*.

[2] Bollen AM, Huang G, King G, Hujoel P, Ma T (2003). Activation time and material stiffness of sequential removable orthodontic appliances. Part 1: ability to complete treatment. *American Journal of Orthodontics and Dentofacial Orthopedics*.

[3] Clements KM, Bollen AM, Huang G, King G, Hujoel P, Ma T (2003). Activation time and material stiffness of sequential removable orthodontic appliances. Part 2: dental improvements. *American Journal of Orthodontics and Dentofacial Orthopedics*.

[4] Kravitz ND, Kusnoto B, BeGole E, Obrez A, Agran B (2009). How well does Invisalign work? A prospective clinical study evaluating the efficacy of tooth movement with Invisalign. *American Journal of Orthodontics and Dentofacial Orthopedics*.

[5] Duong T, Kuo E (2006). Finishing with invisalign. *Progress in orthodontics.*.

[6] McGorray SP, Dolce C, Kramer S, Stewart D, Wheeler TT (2012). A randomized, placebo-controlled clinical trial on the effects of recombinant human relaxin on tooth movement and short-term stability. *American Journal of Orthodontics and Dentofacial Orthopedics*.

[7] Nair MK, Seyedain A, Webber RL (2001). Fractal analyses of osseous healing using tuned aperture computed tomography images. *European Radiology*.

[8] Hua Y, Nackaerts O, Duyck J, Maes F, Jacobs R (2009). Bone quality assessment based on cone beam computed tomography imaging. *Clinical Oral Implants Research*.

[9] Fleiss JL (1985). *The Design and Analysis of Clinical Experiments*.

[10] Fleiss JL, Chilton NW (1983). The measurement of interexaminer agreement on periodontal disease. *Journal of Periodontal Research*.

[11] Krishnan V, Davidovitch Z (2006). Cellular, molecular, and tissue-level reactions to orthodontic force. *American Journal of Orthodontics and Dentofacial Orthopedics*.

[12] Vardimon AD, Robbins D, Brosh T (2010). In-vivo von Mises strains during Invisalign treatment. *American Journal of Orthodontics and Dentofacial Orthopedics*.

[13] Krishnan V, Davidovitch Z (2006). The effect of drugs on orthodontic tooth movement. *Orthodontics & Craniofacial Research*.

[14] Burstone CJ, Pryputniewicz RJ, Weeks R (1981). Centers of resistance of the human mandibular molars. *Journal of Dental Research*.

